# High consumption of fruit and vegetable juices and related beverages and high sugar intake among school-aged children: a study in Beijing, China

**DOI:** 10.3389/fnut.2026.1856451

**Published:** 2026-07-02

**Authors:** Liyu Huang, Lulu Meng, Jiali Duan, Ruoxiang Cao, Manning Wang, Yiran Li, Siyu Liang, Wenjia Li, Huan Luo, Yan Zhang

**Affiliations:** 1 Institute of Nutrition and Food Hygiene, Beijing Center for Disease Prevention and Control, Beijing, China; 2 School of Public Health, Capital Medical University, Beijing, China; 3 Office of Research and Teaching Administration, Beijing Center for Disease Prevention and Control, Beijing, China; 4 School of Public Health, Hebei Medical University, Shijiazhuang, Hebei, China; 5 Xingtai Xindu District Center for Disease Control and Prevention, Xingtai, Hebei, China; 6 Institute of Occupational Health, Beijing Center for Disease Prevention and Control, Beijing, China; 7 Fengtai District Center for Disease Prevention and Control (Fengtai District Health Supervision Institute), Beijing, China

**Keywords:** fruit and vegetable beverages, fruit and vegetable juices, consumption, school-aged children, sugar intake

## Abstract

**Background:**

Fruit and vegetable juices and related beverages are commonly consumed by school-age children, which often contain high levels of free sugars. This study aimed to investigate the consumption and excessive sugar intake from fruit and vegetable juices and related beverages among children in Beijing, and identify associated factors.

**Methods:**

Data were derived from a baseline survey of 3,370 school-age children in Beijing (October–November 2024). Binary logistic regression was used to analyze risk factors for high sugar intake.

**Results:**

The overall consumption rate of fruit and vegetable juices and related beverages among Beijing school-aged children was 68.4%. Overall, the median daily intake of fruit and vegetable juices and related beverages was 57.14 g (0.00, 150.00 g), with a median free sugar intake of 6.06 g (0.00, 15.90 g),15.4% had excessive intake (≥25 g/day). Associations were observed between excessive sugar intake from fruit and vegetable juices and related beverages and the following factors. Living in outer urban areas (*OR* = 1.464, 95%*CI*: 1.140–1.879) or suburban areas (*OR* = 1.443, 95%*CI*: 1.126–1.849), attending middle school (*OR* = 1.936, 95%*CI*: 1.493–2.511) or high school (*OR* = 1.423, 95%*CI*: 1.085–1.868), being overweight (*OR* = 1.345, 95%*CI*: 1.046–1.730), having daily outdoor activity of no less than 2 h (*OR* = 1.348, 95%*CI*: 1.063–1.711), holding the view that sugar-sweetened beverages pose no health risks (*OR* = 1.916, 95%*CI*: 1.561–2.352), and having caregivers who regularly consumed fruit and vegetable juices and related beverages (*OR* = 1.716, 95%*CI*: 1.404–2.097) were all correlated with elevated odds of excessive sugar intake. Among consuming children, the median daily intake of juice beverages and related beverages was 107.14 g (57.14, 214.29 g), with a median free sugar intake of 11.36 g (6.06, 22.71 g), 22.5% had excessive intake (≥25 g/day). Further analyses identified the following correlates for excessive sugar intake in this subgroup: residence in outer urban areas (*OR* = 1.467, 95%*CI*: 1.138–1.892) or suburban areas (*OR* = 1.426, 95%*CI*: 1.108–1.837), male gender (*OR* = 1.289, 95%*CI*: 1.046–1.589), middle school attendance (*OR* = 1.643, 95%*CI*: 1.254–2.152), overweight status (*OR* = 1.341, 95%*CI*: 1.031–1.743), daily outdoor activity of no less than 2 h (*OR* = 1.297, 95%*CI*: 1.011–1.664), the perception of sugar sweetened beverages to health as harmless (*OR* = 1.900, 95%*CI*: 1.532–2.358), and caregivers' consumption of fruit and vegetable juices and related beverages (*OR* = 1.261, 95%*CI*: 1.021–1.556).

**Conclusion:**

High consumption of fruit and vegetable juices and related beverages contributed substantially to free sugar overload among Beijing school-aged children. Targeted nutrition education and family-oriented interventions are urgently needed.

## 1 Introduction

Excessive added sugar intake among children and adolescents has become a major global public health issue, with well-documented associations with adverse health outcomes such as overweight, obesity, dental caries, and metabolic dysfunction (1–3). Fruit and vegetable juices and related beverages (FVJB) can be broadly categorized into two groups. The first group includes fruit and vegetable juices, which are prepared from fruits or vegetables via physical methods (e.g., single-strength juices, reconstituted juices from concentrate, not-from-concentrate (NFC) juices, and concentrated juices). Although these products contain no additional added sugars, they are high in free sugars. The second group comprises fruit and vegetable beverages, which are made from concentrated juices and water, often with high levels of added sugars, acids, flavorings, vitamins, or minerals to improve taste and sensory properties; most palatable fruit and vegetable beverages contain added sugars, sweeteners, acids, and flavorings (4). Evidence indicates that fruit and vegetable juices and beverages are neither superior to nor equivalent to whole fruits and vegetables, nor can they replace plain water (5). Chewing whole fruits results in slow sugar release and absorption over approximately 2 h, whereas the juicing process for fruit and vegetable juices leads to substantial losses of vitamin C and dietary fiber. Processing removes most dietary fiber from fruit and vegetable juices and beverages. Though key nutrients including vitamin C, folate, potassium and carotenoids are readily absorbed, the high sugar content drives rapid blood glucose spikes and brings adverse health effects. Long-term consumption is significantly associated with an increased risk of overweight and obesity in children, reduced gut microbiota diversity, elevated pro-inflammatory factors, and gut microbiota dysbiosis, which can further impair metabolic health, immune function, and cognitive development in school-aged children (5–7). Therefore, school-aged children need to carefully select juice products based on their nutritional needs.

Fruit and vegetable beverages, which are formulated from concentrated juices and water, frequently contain high levels of added sugars (Added sugars belong to free sugars), sweeteners, and other flavor enhancers and are therefore classified as sugar-sweetened beverages (4). International consensus recommends minimizing or avoiding such products to reduce excessive sugar exposure. Notably, pure fruit and vegetable juices contain no added sugars but naturally occurring sugars from raw ingredients, whose levels vary by produce type. Studies have shown that 200 mL of pure juice contains roughly 16 to 24 grams of sugar (8). Current health debates over these juices primarily focus on their high sugar content (5–8). The WHO categorizes sugars in fruit and vegetable juices as free sugars and recommends that free sugar intake account for less than 10% of total daily energy intake (approximately < 50 g/day), with further reduction to below 5% (approximately < 25 g/day) associated with additional health benefits (9).

The 2015–2020 Dietary Guidelines for Americans (DGA) recommended a daily fruit intake of 2 cup-equivalents, with 1 cup of 100% fruit juice counted as 1 cup-equivalent of fruit (10). The DGA explicitly stated that while juice can be part of a healthy dietary pattern, it is lower in dietary fiber than whole fruit and may contribute excess calories when consumed in excess; therefore, at least half of recommended fruit intake should come from whole fruits (10). The updated 2025–2030 DGA further advises limited intake of 100% fruit or vegetable juices or dilution with water (11). Surveys from the United States, the United Kingdom, and Brazil indicate that approximately 34%, 37%, and 42% of respondents reported consuming 100% fruit juice on at least one survey day, with average daily intakes of 184 g, 130 g, and 249 g, respectively (12). Data from the 2015–2017 China National Nutrition and Health Surveillance show that the weekly consumption rate of pure fruit and vegetable juices among Chinese school-aged children was 9.6%, lower than the 15.8% rate for fruit and vegetable beverages (13). In fact, fruit and vegetable beverages ranked third in weekly consumption frequency among children, following carbonated drinks (22.8%) and milk-containing beverages (19.1%) (13). In practice, many school-aged children and their caregivers lack awareness of the health risks posed by high free sugar levels in fruit and vegetable juices. Furthermore, they often fail to distinguish pure juices from juice beverages, which typically contain high added sugars and additives but offer more appealing flavors; products labeled with fruit or vegetable images or text are frequently misperceived as healthy choices. Accordingly, to ensure adequate nutrient intake while avoiding excessive free or added sugar consumption, the Chinese Dietary Guidelines (2022) strongly recommends that school-aged children consume whole fruits and vegetables instead of fruit and vegetable juices and related beverages (14).

With advances in socioeconomic development, food processing, preservation, and related technologies, the food environment, dietary variety, consumption levels, and dietary habits and preferences of school-aged children have undergone substantial changes. However, evidence remains scarce in mainland China regarding the consumption patterns of fruit and vegetable juices and related beverages among school-aged children, the corresponding levels of sugar intake, and the associated demographic, cognitive, and behavioral factors. As the capital city of China, Beijing provides widespread access to a broad range of beverages among local children. Nevertheless, empirical data remain limited on the consumption of fruit and vegetable juices and related beverages, the associated free sugar and added sugar intakes, and the influencing factors related to excessive sugar consumption. This study therefore aims to investigate the consumption of fruit and vegetable juices and related beverages among school-aged children in Beijing, quantify their intake of free and added sugars, and identify factors linked to excessive sugar consumption. Drawing on existing literature and theoretical frameworks, we selected demographic, cognitive, behavioral and household factors as key variables. Previous studies have consistently confirmed that these factors are closely associated with children's beverage choices and sugar intake, and they are also feasible to measure and applicable to the local Chinese context. Our *a priori* hypotheses regarding the relationships between these factors and excessive sugar intake are presented as follows: 1) Demographic hypotheses: Residential area, gender and school grade are correlated with children's exposure to high-sugar beverages. Specifically, children whose caregivers have higher educational attainment tend to have a lower risk of high sugar intake. Differences in the accessibility of sugary drinks across groups may also lead to disparities in sugar consumption levels. 2) Cognitive hypotheses: Children and families with better awareness of the health risks of sugar intake and greater attention to food labels are less likely to consume high-sugar beverages. Conversely, poor knowledge about sugar-related health hazards and limited understanding of food labels may increase the likelihood of excessive sugar intake. In addition, misconceptions that fruit and vegetable juices can replace whole fruits and vegetables may indirectly raise sugar intake risks. 3) Behavioral hypotheses: Daily outdoor activity duration is strongly associated with sugar exposure, and this behavioral factor can be effectively modified via environmental and educational interventions. 4) Household-related hypotheses: Family size, caregivers' educational level, caregivers' perceptions of whether fruit and vegetable juices can substitute for whole fruits and vegetables, and caregivers' own consumption of such juices are all correlated with excessive sugar intake among school-aged children. The findings of this study will provide evidence to support the development of targeted interventions and public health policies for curbing excessive sugar intake and improving overall dietary quality among children.

## 2 Materials and methods

### 2.1 Study population

The data used in this study were derived from the baseline data of a cohort study on sugar-sweetened beverage consumption behavior among primary and secondary school students, which was initiated in October 2024 in Beijing, China. Specifically, this baseline data were collected through a cross-sectional survey conducted in Dongcheng District, Fengtai District, and Daxing District of Beijing between October and November 2024. The study population comprised school-aged children who participated in the consumption of fruit and vegetable juices and related beverages component of the baseline survey and for whom the relevant data were complete. Participants were selected by inclusion criteria: (1)Primary and secondary school students enrolled in Beijing, China; (2) The student and their primary caregiver voluntarily participate and sign an informed consent form; (3) Ability to understand the questionnaire content and complete it independently. Exclusion Criteria: (1) Students who are currently on a leave of absence due to illness or other reasons; (2) Students who are unable to understand or complete the questionnaire; (3) Students who refuse to participate.

A multi-stage stratified cluster sampling design was employed. Beijing was divided into three residence areas (central urban area, outer urban area, and suburban area) based on population, geographic location, and levels of economic and cultural development. To ensure sample representativeness, the sample size was calculated based on the 2022 consumption rate of fruit and vegetable juices among school-age children in Beijing, with a proportion (*p*) of 0.323 (15). The required sample size was computed using the formula *N* = $\mu_{\text{α}/2}^{2}\times$ *p*×(1-*p*)/δ^2^× *deff* , where μ_α/2_ = 1.96 for a 95% two-sided confidence level, the margin of error δ = 0.15 × *p* = 0.0975, and the design effect deff = 3. Anticipating a 10% rate of non-response/invalid questionnaires, the minimum sample size for the overall estimate is about 295. The study employed stratified sampling across core urban, distant urban, and suburban districts. Accordingly, the minimum total sample size for the stratified design was 855. Across the cohort, 3,514 school-aged children participated in the baseline survey. Among them, 3,370 had complete data on demographics, height, weight, consumption patterns of fruit and vegetable juices and related beverages, yielding an effective sample size of 3,370 and an effective response rate of 95.9%. The effective sample size of 3,370 far exceeds the stated minimum of 855, indicating that the sample has ample representativeness.

Data were collected using a mixed approach of interviewer-administered and self-administered questionnaires. For primary school students, interviews were primarily conducted face-to-face by investigators at school. For middle and high school students, surveys were mainly self-administered. The study was approved by the Ethics Committee of the Beijing Center for Disease Prevention and Control (BJCDC2024031). Written informed consent was obtained from the students' caregivers and from the participating students prior to the study.

### 2.2 Data collection

Two self-designed questionnaires were used in the survey: a sugar-sweetened beverage consumption behavior questionnaire for school-aged children and a family environment questionnaire. These questionnaires were developed based on the Chinese Dietary Guidelines (2022), the Encyclopedia of Nutrition Science, and other literature (14, 16, 24), and refined through focused group discussions, expert review, and repeated pretesting. Invited experts with rich experience and professional knowledge in related fields, including epidemiologists, public health experts, nutritionists, and school health education teachers, to review and evaluate the questionnaire. Based on the experts' revision suggestions, ensure that the questionnaire has good content validity and scientificity. The Cronbach's alpha coefficients were 0.81 and 0.75, respectively, indicating good reliability. The questionnaires mainly covered basic demographic information, beverage consumption-related information, health related perception and behavioral information. The sugar-sweetened beverage consumption behavior questionnaire and the family environment questionnaire were completed independently by the students and their parents, respectively. Student questionnaires were administered in school under teacher supervision, while caregivers questionnaires were completed at home. The responses from students and caregivers were matched one-to-one via questionnaire codes.

#### 2.2.1 Demographic information

Demographic characteristics included gender, age, grade, residence areas, and caregiver's education level. Grade level of school-aged children was defined as primary school (Grade 3), middle school (Grade 7), and high school (Grade 10). Residence areas were categorized as central urban, outer urban, and suburban areas.

#### 2.2.2 Assessment of consumption patterns of fruit and vegetable juices and related beverages

Consumption of fruit and vegetable juices and beverages was evaluated according to consumption frequency and average serving volume in the past week. These products were classified into fruit and vegetable juices and fruit and vegetable beverages. Four questions were used to assess consumption frequency, with response options including times per day, times per week, and almost never (0 times/week). Participants also reported their average serving size (mL) per occasion. All consumption frequencies were converted to times per week for unified analysis.

Consumption was defined as intake at least once in the past week, and the consumption rate was calculated as the proportion of consumers to the total surveyed students multiplied by 100%.

Daily intake of fruit and vegetable juices and beverages was calculated by dividing their total weekly consumption (determined from weekly intake frequency and average serving size) by seven. A previous study by Deng et al. from the China National Center for Food Safety Risk Assessment measured sugar levels in commercial beverages across China, reporting that fruit and vegetable juices and juice drinks contained 10.6 g of sugar per 100 mL (17). We adopted this sugar concentration to estimate free sugar intake from the above beverages in our study. Specifically, daily free sugar intake was computed as total daily beverage volume multiplied by 10.6 g/100 mL.

The WHO categorizes sugars in fruit and vegetable juices as free sugars and recommends that free sugar intake account for less than 10% of total daily energy intake (approximately < 50 g/day), with further reduction to below 5% (approximately < 25 g/day) associated with additional health benefits (9). The Chinese Dietary Guidelines (2022) issued by the Chinese Nutrition Society suggests that daily free sugar intake for school-age children should ideally be controlled below 25 g (14). Taking into account China's geographical, demographic, economic, cultural and dietary factors, this study defined a daily free sugar intake of ≥25 g from fruit and vegetable juices and related beverages as the cutoff for high-risk free sugar exposure among school-age children.

#### 2.2.3 Perceptions and health-related behaviors associated with fruit and vegetable juices and related beverages

This section assessed school-aged children's perceptions of fruit and vegetable juices and related beverages, their practice of checking food labels before purchase, and their daily outdoor activity behaviors using the following four questions. Question 1: Do you believe fruit and vegetable juice can replace whole fruits and vegetables? Response options: Yes; No. Question 2: Do you think sugar-sweetened beverages are harmful to health? Response options: Not harmful; Harmful. Question 3: Do you check food labels before purchasing food items? Response options: Never check; Check. Question 4: In the past week, what was your average daily duration of daytime outdoor activity? Response options: < 0.5 h; 0.5–1 h; 1–2 h; ≥2 h. Outdoor activity was defined as any physical activity carried out outdoors, including outdoor physical education classes, extracurricular sports, walking, cycling, and other related activities. For school-aged children, an average daily daytime outdoor activity duration of ≥2 h was defined as meeting the recommended level, whereas < 2 h was defined as insufficient (18).

#### 2.2.4 Health-related indicators

Weight status was assessed using body mass index (BMI), calculated as weight in kilograms divided by height in meters squared (kg/m^2^). Height and weight measurements for school-age children were obtained from the schools' routine physical examination records. In accordance with Screening Standard for Malnutrition in School-Age Children and Adolescents (WS/T 456-2014), wasting was defined as a BMI below the age- and sex-specific reference cutoffs (19). According to Screening for Overweight and Obesity among School-Age Children and Adolescents (WS/T 586-2018), overweight was defined as a BMI at or above the age- and sex-specific overweight threshold but below the obesity threshold, while obesity was defined as a BMI at or above the corresponding obesity threshold (20). These two national standards are applicable for screening wasting, overweight, and obesity among school-age children of all ethnic groups across China, and were both issued and recommended for implementation by the National Health Commission of the People's Republic of China. Data on dental caries and myopia among school-age children were also obtained from their routine physical examination records (21, 22).

#### 2.2.5 Family-related factors

In this study, family-related variables included caregiver educational attainment (high school or below; college or above), the number of children in the household (1; ≥2), caregivers' perceptions of whether fruit and vegetable juices and related beverages could replace whole fruits and vegetables (replaceable; non-replaceable), and caregivers' consumption of fruit and vegetable juices and related beverages (non-consumption; consumption).

### 2.3 Quality control

A three-tier quality control system was established at the project team, project sites, and survey schools. Investigators underwent standardized training and were deemed qualified upon assessment. During field surveys, procedures were strictly carried out in accordance with the survey protocol. On-site quality control personnel promptly identified and corrected omissions, logical errors, and other issues detected in the field. After the survey, data were entered into a database using EpiData 3.1 by professionally trained personnel, with double data entry used to input, verify, and clean the survey data.

### 2.4 Statistical analysis

Statistical analyses and descriptive statistics were performed using SPSS 21.0. As daily intake of fruit and vegetable juices and related beverages and corresponding daily free/added sugar intake were quantitative data not normally distributed, they were presented as medians with interquartile ranges [*M* (*P*_25_, *P*_75_)]. Between-group differences were analyzed using non-parametric tests: the Mann-Whitney U test for two groups and the Kruskal-Wallis H test for three or more groups. Categorical data were described using frequencies (n) and percentages (%), with between-group comparisons conducted via the chi-square test. A binary logistic regression model was employed to identify factors associated with excessive daily free sugar intake (≥25 g) among school-age children. Daily excessive free sugar intake (≥25 g) was set as the dependent variable (1 = ≥ 25 g/day; 0 = < 25 g/day). Independent variables entered into the multivariable logistic regression model included all factors with statistically significant differences in univariate analysis: residential area (central urban = 0; outer urban = 1; suburban = 2), gender (girls = 0; boys = 1), grade (primary school = 0; middle school = 1; high school = 2), weight status (normal = 0; underweight = 1; overweight = 2; obese = 3), daily outdoor activity time (0 = < 2 h/day; 1 = ≥ 2 h/day), perception of harm from sugar-sweetened beverages (0 = harmless;1 = harmful), caregiver education level (0 = high school or below; 1 = college or above), and caregiver consumption of fruit and vegetable juices and related beverages (0 = non-consumption; 1 = consumption). A significance level of α = 0.05 was used for all statistical tests.

## 3 Results

### 3.1 Baseline characteristics

The general characteristics of the study participants are summarized in Table 1. In terms of residential distribution, 34.7% lived in central urban area, 31.9% in outer urban area, and 33.4% in suburban area. By gender, boys accounted for 50.6% and girls 49.4%. Regarding school stage, primary school students comprised 31.1%, junior high school students 34.7%, and senior high school students 34.1%. For ethnicity, 91.4% were of Han nationality, with the remaining 8.6% belonging to other ethnic groups. Household registration was urban in 88.6% of cases and agricultural in 11.4%. In terms of weight status, 55.3% of children had normal weight, 6.4% were underweight, 17.9% were overweight, and 20.5% were obese. Regarding health conditions, 32.7% had dental caries, while 67.3% did not; 68.8% had myopia, compared with 31.2% without myopia. Concerning health perceptions and behaviors, 74.1% of children considered sugar sweetened beverages harmful to health, whereas 25.9% did not. Most participants (89.8%) believed fruit and vegetable juices and related beverages could not replace whole fruits and vegetables, while 10.2% held the opposite view. When purchasing food, 58.8% reported checking food labels, compared with 41.2% who did not. For daily outdoor activity, 82.9% engaged in less than 2 h per day, and only 17.1% achieved 2 h or more. Family-related characteristics showed that 60.9% of children were from single-child families, and 39.1% from families with two or more children. Caregivers' education level was college or above in 62.7% of cases and high school or below in 37.3%. The vast majority of caregivers (97.6%) did not regard fruit and vegetable juices and related beverages as a substitute for whole fruits and vegetables. In terms of consumption behavior, 55.4% of caregivers consumed fruit and vegetable juices and related beverages, while 44.6% did not.

**Table 1:** Characteristics of the participants.

Characteristics	Frequency (*n*)	Percentage (%)
Total	3,370	100.0
Residence area		
Central urban	1,170	34.7
Outer urban	1,076	31.9
Suburban	1,124	33.4
Gender		
Boys	1,705	50.6
Girls	1,665	49.4
Grade		
Primary school	1,049	31.1
Middle school	1,171	34.7
High school	1,150	34.1
Ethnicity		
Han	3,081	91.4
Others	289	8.6
Household registration type		
Agricultural	383	11.4
Urban	2,987	88.6
Weight type status		
Normal	1,863	55.3
Underweight	214	6.4
Overweight	603	17.9
Obese	690	20.5
Dental caries		
No	2,269	67.3
Yes	1,101	32.7
Myopia		
No	1,051	31.2
Yes	2,319	68.8
Perception of sugar sweetened beverages harm to health		
Harmless	872	25.9
Harmful	2,498	74.1
Perception of fruit and vegetable juices and related beverages replacing whole fruits and vegetables		
Replaceable	344	10.2
Non-replaceable	3,026	89.8
Checking food labels before purchase		
Never check	1,390	41.2
Check	1,980	58.8
Daily outdoor activity time		
< 2 h/day	2,795	82.9
≥2 h/day	575	17.1
Number of children in family		
≥2	1,319	39.1
1	2,051	60.9
Caregiver's education		
High school or below	1,258	37.3
College or above	2,112	62.7
Caregivers' perception of fruit and vegetable juices and related beverages replacing whole fruits and vegetables		
Replaceable	82	2.4
Non-replaceable	3,288	97.6
Caregivers' consumption of fruit and vegetable juices and related beverages		
Non-consumption	1,504	44.6
Consumption	1,866	55.4

### 3.2 Frequency distribution of consumption among school-aged children

#### 3.2.1 Frequency distribution of fruit and vegetable juices consumption among school-aged children

**Figure 1:**
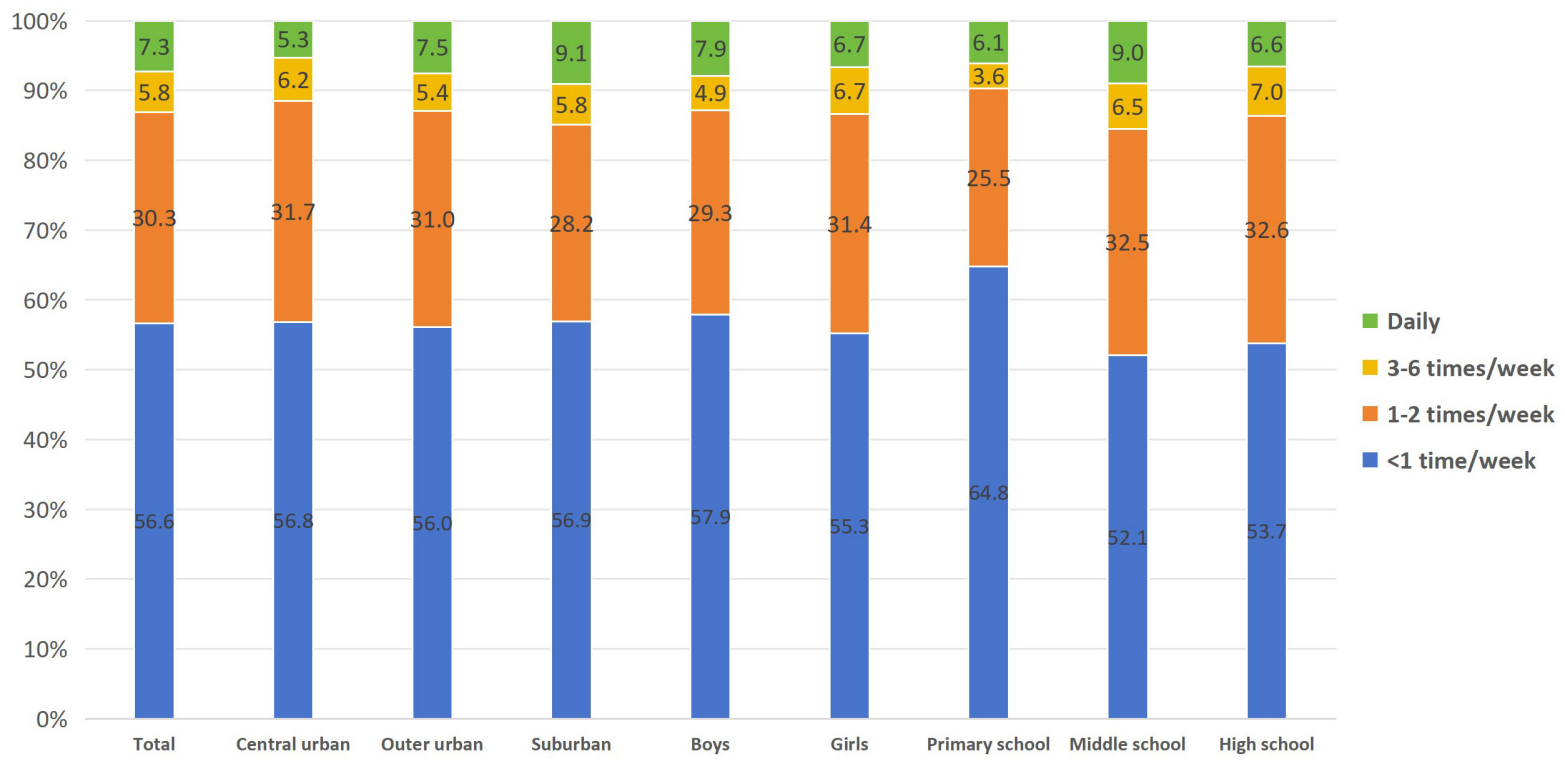
Frequency distribution of fruit and vegetable juices consumption among school-aged children in different residence areas, genders and grades.

Figure 1 illustrates the consumption frequency distribution of fruit and vegetable juices among school-aged children. The percentages of children consumed fruit and vegetable juices < 1 time/week, 1–2 times/week, 3–6 times/week, daily, at 50.6%, 30.3%, 5.8%, and 7.3%, respectively. Differences in the frequency distribution of fruit and vegetable juices consumption among school-age children defined by residence areas (**χ^2^** = 14.634, *p* = 0.023), gender (**χ^2^** = 8.411, *p* = 0.038) and grade (**χ^2^** = 50.261, *p* < 0.001) were statistically significant.

#### 3.2.2 Frequency distribution of fruit and vegetable beverages consumption among school-aged children

**Figure 2:**
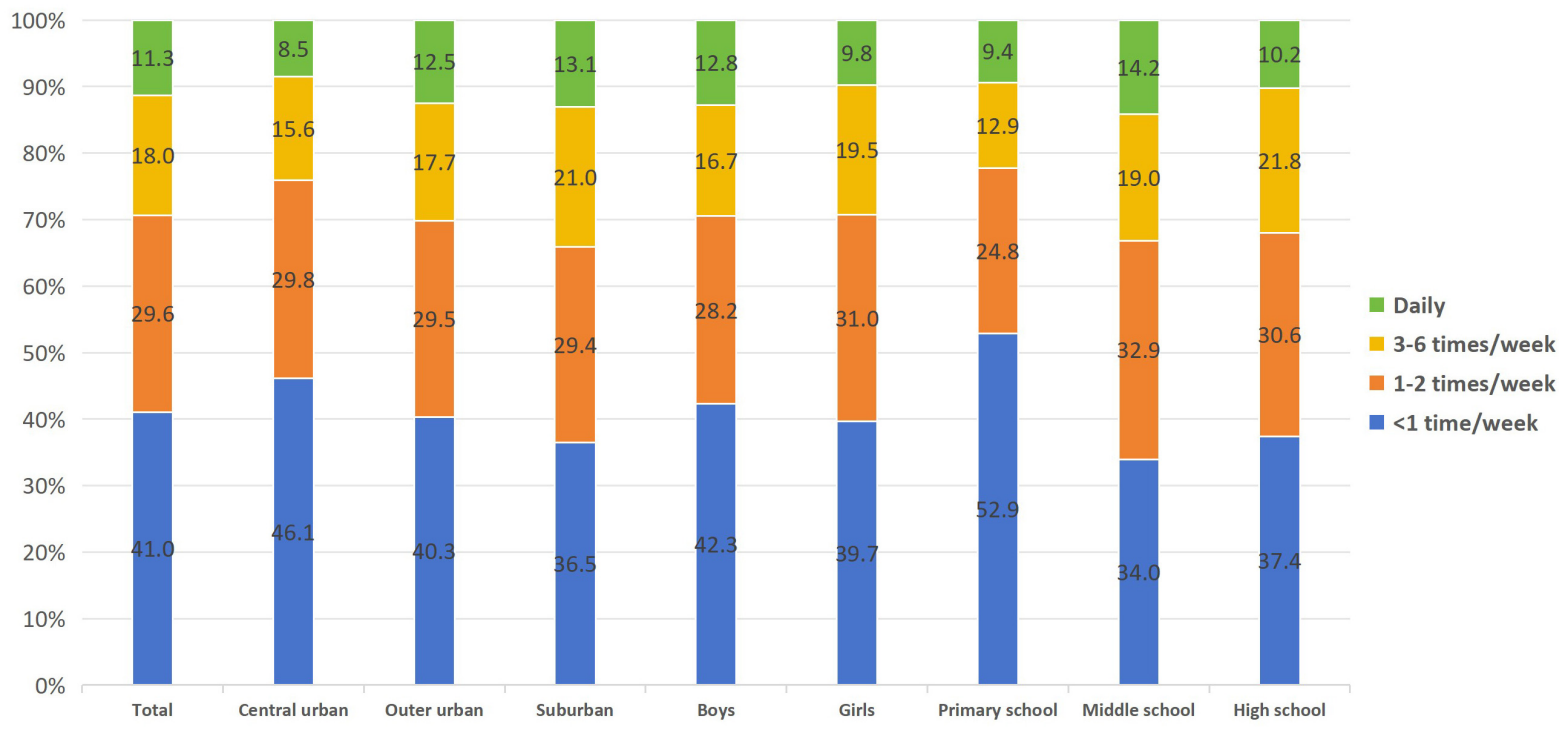
Frequency distribution of fruit and vegetable beverages consumption among school-aged children in different residence areas, genders and grades.

Figure 2 illustrates the consumption frequency distribution of fruit and vegetable beverages among school-aged children. The percentages of children consumed fruit and vegetable beverages < 1 time/week, 1–2 times/week, 3–6 times/week, daily, at 41.0%, 29.6%, 18.0%, and 11.3%, respectively. Differences in the frequency distribution of fruit and vegetable beverages consumption among school-aged children defined by residence areas (**χ^2^** = 35.043, *p* < 0.001), gender (**χ^2^** = 13.711, *p* = 0.003) and grade (**χ^2^** = 105.054, *p* < 0.001) were statistically significant.

#### 3.2.3 Frequency distribution of fruit and vegetable juices and related beverages consumption among school-aged children

**Figure 3:**
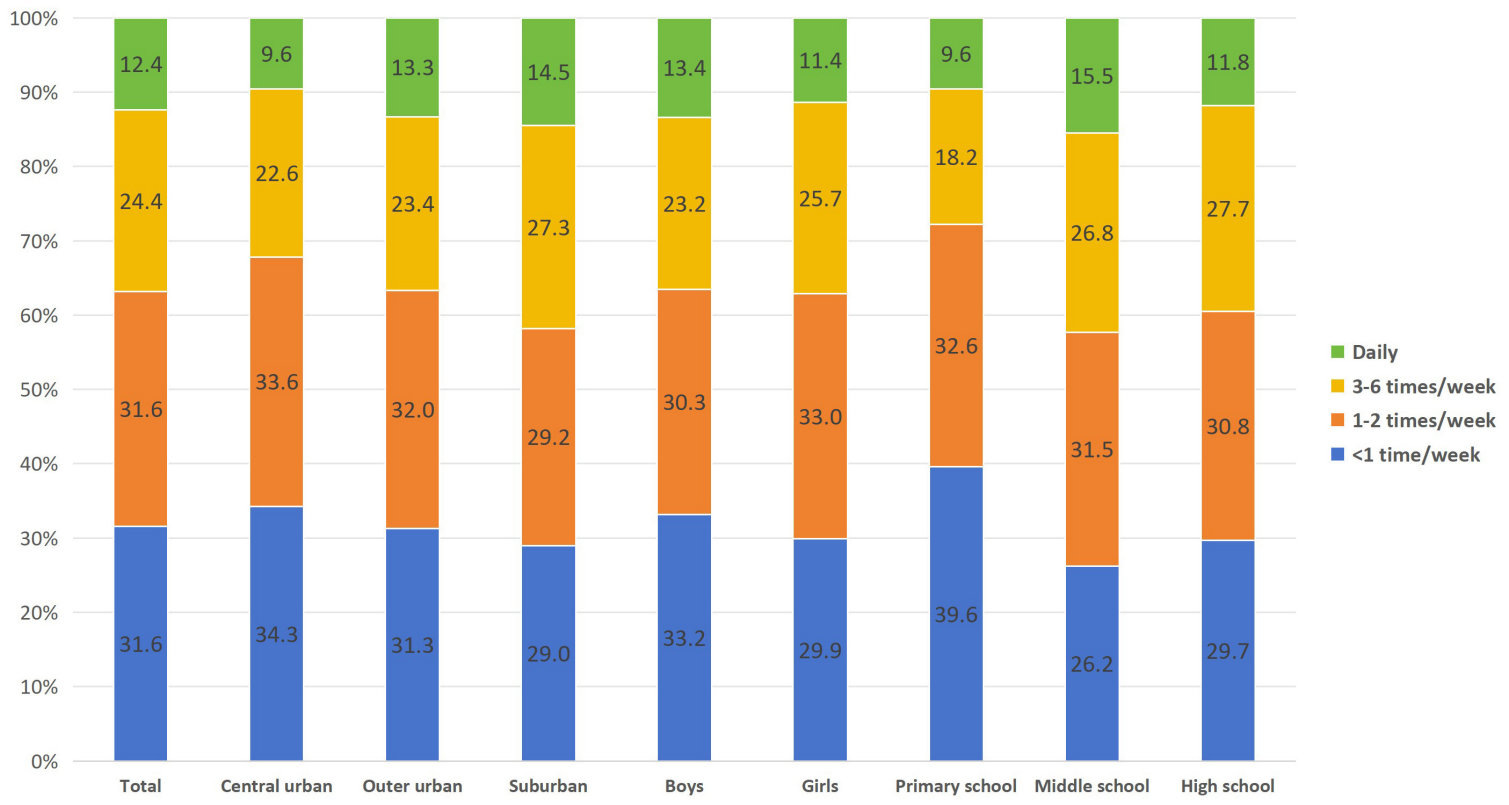
Frequency distribution of fruit and vegetable juices and related beverages consumption among school-aged children in different residence areas, genders and grades.

Figure 3 illustrates the consumption frequency distribution of fruit and vegetable juices and related beverages among school-aged children. Overall, the percentages of children consumed these products < 1 time/week, 1–2 times/week, 3–6 times/week, daily, at 31.6%,31.6%, 24.4%, and 12.4%, respectively. Differences in the frequency distribution of fruit and vegetable juices and related beverages consumption among school-aged children defined by residence areas (**χ^2^**= 26.841, *p* < 0.001), gender (**χ^2^**= 9.673, *p* =0.022) and grade (**χ^2^** = 73.51, *p* < 0.001) were statistically significant.

### 3.3 Consumption of fruit and vegetable juices and related beverages among school-aged children

This study primarily aimed to examine consumption of fruit and vegetable juices and related beverages among school-aged children and assess the health risks associated with consequent excessive free sugar intake. Accordingly, consumption of fruit and vegetable juices and related beverages was combined for subsequent in-depth analysis and evaluation. In this study, consumption was defined as intake fruit and vegetable juices and related beverages at least once in the past week, and the consumption rate was calculated as the proportion of consumers to the total surveyed students multiplied by 100%. As shown in the Table 2, the overall consumption rate of fruit and vegetable juices and related beverages among school-age children was 68.4%. Significant differences were found by residence area, gender, grade, myopia, perceived harm of sugar-sweetened beverages, daily outdoor activity time, and caregiver's education (all *p* < 0.05). Higher consumption rates were observed in suburban areas, among girls, middle school students, children with myopia, those perceiving sugar sweetened beverages as harmless, those with ≥2 h of outdoor activity, and those whose caregivers had lower education levels. Caregivers' fruit and vegetable juices and related beverages consumption was strongly associated with children's consumption (77.3% vs. 57.4%, *p* < 0.001). No significant differences were observed for ethnicity, household registration, weight status, dental caries, related cognitive attitudes, food label checking, or number of children in the family (all *p* > 0.05).

**Table 2:** Consumption of fruit and vegetable juices and related beverages among school-aged children in Beijing, China.

Characteristics	Consumption rate, *n*(%)	χ^2^	*P*-value
Total	2,306 (68.4)		
Residence area		7.416	0.025
Central urban	769 (65.7)		
Outer urban	739 (68.7)		
Suburban	798 (71.0)		
Gender		4.212	0.040
Boys	1,139 (66.8)		
Girls	1,167 (70.1)		
Grade		48.325	< 0.001
Primary school	634 (60.4)		
Middle school	864 (73.8)		
High school	808 (70.3)		
Ethnicity		0.482	0.488
Han	2,103 (68.3)		
Others	203 (70.2)		
Household registration type		0.683	0.409
Agricultural	255 (66.6)		
Urban	2,051 (68.7)		
Weight type status		2.675	0.444
Normal	1,268 (68.1)		
Underweight	148 (69.2)		
Overweight	428 (71.0)		
Obese	462 (67.0)		
Dental caries		0.072	0.789
No	1,556 (68.6)		
Yes	750 (68.1)		
Myopia		5.446	0.020
No	690 (65.7)		
Yes	1,616 (69.7)		
Perception of sugar sweetened beverages harm to health		8.432	0.004
Harmless	631 (72.4)		
Harmful	1,675 (67.1)		
Perception of fruit and vegetable juices and related beverages replacing whole fruits and vegetables		0.435	0.509
Replaceable	230 (66.9)		
Non-replaceable	2,076 (68.6)		
Checking food labels before purchase		2.299	0.129
Never check	931 (67.0)		
Check	1,375 (69.4)		
Daily outdoor activity time		6.333	0.012
< 2 h/day	1,887 (67.5)		
≥2 h/day	419 (72.9)		
Number of children in family		1.203	0.273
≥2	917 (69.5)		
1	1,389 (67.7)		
Caregiver's education		6.081	0.014
High school or below	893 (71.0)		
College or above	1,413 (66.9)		
Caregivers' perception of fruit and vegetable juices and related beverages replacing whole fruits and vegetables		0.875	0.349
Replaceable	60 (73.2)		
Non-replaceable	2,246 (68.3)		
Caregivers' consumption of fruit and vegetable juices and related beverages		151.589	< 0.001
Non-consumption	864 (57.4)		
Consumption	1,442 (77.3)		

### 3.4 Consumption characteristics of fruit and vegetable juices and related beverages in the total study population

#### 3.4.1 Daily intake of fruit and vegetable juices and related beverages and corresponding free sugar intake in the total study population

Table 3 presents the daily intake of fruit and vegetable juices and related beverages and corresponding free sugar intake among school-age children in Beijing, China. Overall, the median daily intake of fruit and vegetable juices and related beverages was 57.14 g (0.00, 150.00 g), with a median free sugar intake of 6.06 g (0.00, 15.90 g). Residence area, grade, ethnicity, myopia, perceived harm of sugar sweetened beverages, daily outdoor activity time, caregiver's education level, and caregiver's fruit and vegetable juices and related beverages consumption were all significantly associated with both fruit and vegetable juices and related beverages intake and free sugar intake (all *p* < 0.05). In particular, children living in suburban areas, those in middle school, children with myopia, those perceiving sugar sweetened beverages as harmless, those with ≥2 h of daily outdoor activity, and those whose caregivers had lower educational levels showed higher intake levels. Non-Han children also had higher intake than Han children. Notably, children whose caregivers consumed fruit and vegetable juices and related beverages had markedly higher intake of fruit and vegetable juices and related beverages (71.43 g, 14.29–200.00 g) and free sugar (7.57 g, 1.51–21.20 g) than those whose caregivers did not (28.57 g, 0.00–121.43 g; 3.03 g, 0.00–12.87 g). In contrast, no significant differences were observed in intake levels by gender, household registration type, weight status, dental caries, perception of fruit and vegetable juices and related beverages replacing whole fruits and vegetables, food label checking behavior, number of children in the family, or caregivers' perception of fruit and vegetable juices and related beverages replacing whole fruits and vegetables (all *p* > 0.05).

**Table 3:** The median daily intake of fruit and vegetable juices and related beverages and median daily free sugar intake from such beverages among school-age children in Beijing, China.

Characteristics	Daily intake of fruit and vegetable juices and related beverages *M*(*P*_25_, *P*_75_)	Daily free sugar intake *M*(*P*_25_, *P*_75_)	χ^2^	*P*-value
Total	57.14 (0.00, 150.00)	6.06 (0.00, 15.90)		
Residence area			25.852	< 0.001
Central urban	42.86 (0.00, 128.57)	4.54 (0.00, 13.63)		
Outer urban	57.14 (0.00, 171.43)	6.06 (0.00, 18.17)		
Suburban	71.43 (0.00, 185.71)	7.57 (0.00, 19.69)		
Gender			−0.207	0.836
Boys	64.29 (0.00, 157.14)	6.81 (0.00, 16.66)		
Girls	57.14 (0.00, 142.86)	6.06 (0.00, 15.14)		
Grade			121.528	< 0.001
Primary school	28.57 (0.00, 95.00)	3.03 (0.00, 10.07)		
Middle school	78.57 (0.00, 190.00)	8.33 (0.00, 20.14)		
High school	71.43 (0.00, 171.43)	7.57 (0.00, 18.17)		
Ethnicity			−2.079	0.038
Han	57.14 (0.00, 142.86)	6.06 (0.00, 15.14)		
Others	71.43 (0.00, 203.57)	7.57 (0.00, 21.58)		
Household registration type			0.627	0.531
Agricultural	71.43 (0.00, 178.57)	7.57 (0.00, 18.93)		
Urban	57.14 (0.00, 142.86)	6.06 (0.00, 15.14)		
Weight type status			5.429	0.143
Normal	57.14 (0.00, 142.86)	6.06 (0.00, 15.14)		
Underweight	57.14 (0.00, 142.86)	6.06 (0.00, 15.14)		
Overweight	71.43 (0.00, 178.57)	7.57 (0.00, 18.93)		
Obese	57.14 (0.00, 142.86)	6.06 (0.00, 15.14)		
Dental caries			−1.099	0.272
No	64.29 (0.00, 157.14)	6.18 (0.00, 16.66)		
Yes	57.14 (0.00, 142.86)	6.06 (0.00, 15.14)		
Myopia			−3.447	0.001
No	42.86 (0.00, 142.86)	4.54 (0.00, 15.14)		
Yes	71.43 (0.00, 157.14)	7.57 (0.00, 16.66)		
Perception of sugar sweetened beverages harm to health			−6.916	< 0.001
Harmless	78.57 (0.00, 214.29)	8.33 (0.00, 22.71)		
Harmful	42.86 (0.00, 142.86)	4.54 (0.00, 15.14)		
Perception of fruit and vegetable juices and related beverages replacing whole fruits and vegetables			−0.706	0.480
Replaceable	42.86 (0.00, 142.86)	4.54 (0.00, 15.14)		
Non-replaceable	57.14 (0.00, 150.00)	6.06 (0.00, 15.90)		
Checking food labels before purchase			−0.107	0.915
Never check	64.29 (0.00, 157.14)	6.81 (0.00, 16.66)		
Check	57.14 (0.00, 142.86)	6.06 (0.00, 15.14)		
Daily outdoor activity time			−3.727	< 0.001
< 2 h/day	57.14 (0.00, 142.86)	6.06 (0.00, 15.14)		
≥2 h/day	71.43 (0.00, 200.00)	7.57 (0.00, 21.2)		
Number of children in family			−0.545	0.585
≥2	57.14 (0.00, 150.00)	6.06 (0.00, 15.90)		
1	57.14 (0.00, 145.71)	6.06 (0.00, 15.45)		
Caregiver's education			−3.696	< 0.001
High school or below	71.43 (0.00, 171.43)	7.57 (0.00, 18.17)		
College or above	50.00 (0.00, 142.86)	5.30 (0.00, 15.14)		
Caregivers' perception of fruit and vegetable juices and related beverages replacing whole fruits and vegetables			−1.725	0.085
Replaceable	71.43 (0.00, 192.50)	7.57 (0.00, 20.41)		
Non-replaceable	57.14 (0.00, 142.86)	6.06 (0.00, 15.14)		
Caregivers' consumption of fruit and vegetable juices and related beverages			−11.634	< 0.001
Non-consumption	28.57 (0.00, 121.43)	3.03 (0.00, 12.87)		
Consumption	71.43 (14.29, 200.00)	7.57 (1.51, 21.20)		

#### 3.4.2 Univariate analysis of excessive daily free sugar intake from fruit and vegetable juices and related beverages in the total study population

Table 4 shows the proportion of excessive daily free sugar intake (≥25 g) from fruit and vegetable juices and related beverages among school-aged children in Beijing. Overall, 518 children (15.4%) had free sugar intake exceeding the recommended level. Univariate analyses identified several factors significantly associated with excessive free sugar intake, including residence area, gender, grade, weight status, perceived harm of sugar-sweetened beverages, daily outdoor activity duration, caregiver's educational level, and caregiver's consumption of fruit and vegetable juices and related beverages (all *p* < 0.05). The proportion of children with excessive free sugar intake was higher in suburban and outer urban areas than in central urban areas. Boys were more likely to have excessive intake than girls. By grade, middle school students had the highest proportion (20.1%), followed by high school students and primary school students. Overweight children showed a higher proportion of excessive intake compared with normal-weight and underweight children. Children who perceived sugar-sweetened beverages as harmless, those with at least 2 h of daily outdoor activity, and those whose caregivers had lower education or consumed fruit and vegetable juices were more likely to have free sugar intake ≥25 g. No significant associations were observed for ethnicity, household registration type, dental caries, myopia, perceptions regarding juice replacing whole fruits and vegetables, food label checking behavior, or number of children in the family (all *p* > 0.05).

**Table 4:** The proportion of excessive daily free sugar intake (≥25 g) from fruit and vegetable juices and related beverages among school-aged children in Beijing, China.

Characteristics	< 25 g/day, *n*(%)	≥25 g/day, *n* (%)	χ^2^	*P*-value
Total	2,852 (84.6)	518 (15.4)		
Residence area			17.859	< 0.001
Central urban	1,032 (88.2)	138 (11.8)		
Outer urban	886 (82.3)	190 (17.7)		
Suburban	934 (83.1)	190 (16.9)		
Gender			6.616	0.010
Boys	1,416 (83.0)	289 (17.0)		
Girls	1,436 (86.2)	229 (13.8)		
Grade			44.791	< 0.001
Primary school	946 (90.2)	103 (9.8)		
Middle school	936 (79.9)	235 (20.1)		
High school	970 (84.3)	180 (15.7)		
Ethnicity			0.072	0.788
Han	2,609 (84.7)	472 (15.3)		
Others	243 (84.1)	46 (15.9)		
Household registration type			1.887	0.169
Agricultural	315 (82.2)	68 (17.8)		
Urban	2,537 (84.9)	450 (15.1)		
Weight type status			10.440	0.015
Normal	1,604 (86.1)	259 (13.9)		
Underweight	184 (86.0)	30 (14.0)		
Overweight	488 (80.9)	115 (19.1)		
Obese	576 (83.5)	114 (16.5)		
Dental caries			2.407	0.121
No	1,905 (84.0)	364 (16.0)		
Yes	947 (86.0)	154 (14.0)		
Myopia			2.250	0.134
No	904 (86.0)	147 (14.0)		
Yes	1,948 (84.0)	371 (16.0)		
Perception of sugar sweetened beverages harm to health			61.596	< 0.001
Harmless	666 (76.4)	206 (23.6)		
Harmful	2,186 (87.5)	312 (12.5)		
Perception of fruit and vegetable juices and related beverages replacing whole fruits and vegetables			0.423	0.515
Replaceable	287 (83.4)	57 (16.6)		
Non-replaceable	2,565 (84.8)	461 (15.2)		
Checking food labels before purchase			0.655	0.418
Never check	1,168 (84.0)	222 (16.0)		
Check	1,684 (85.1)	296 (14.9)		
Daily outdoor activity time			14.140	< 0.001
< 2 h/day	2,395 (85.7)	400 (14.3)		
≥2 h/day	457 (79.5)	118 (20.5)		
Number of children in family			1.947	0.163
≥2	1,102 (83.5)	217 (16.5)		
1	1,750 (85.3)	301 (14.7)		
Caregiver's education			4.151	0.042
High school or below	1,044 (83.0)	214 (17.0)		
College or above	1,808 (85.6)	304 (14.4)		
Caregivers' perception of fruit and vegetable juices and related beverages replacing whole fruits and vegetables			0.552	0.458
Replaceable	67 (81.7)	15 (18.3)		
Non-replaceable	2,785 (84.7)	503 (15.3)		
Caregivers' consumption of fruit and vegetable juices and related beverages			31.245	< 0.001
Non-consumption	1,331 (88.5)	173 (11.5)		
Consumption	1,521 (81.5)	345 (18.5)		

#### 3.4.3 Multivariate binary logistic regression for excessive daily free sugar intake (≥25 g) among school-aged children

Table 5 presents the results of multivariate binary logistic regression for excessive daily free sugar intake (≥25 g) among school-aged children. Associations were observed between excessive sugar intake from fruit and vegetable juices and related beverages and the following factors. Living in outer urban areas (*OR* = 1.464, 95%*CI*: 1.140–1.879) or suburban areas (*OR* = 1.443, 95%*CI*: 1.126–1.849), attending middle school (*OR* = 1.936, 95%*CI*: 1.493–2.511) or high school (*OR* = 1.423, 95%*CI*: 1.085–1.868), being overweight (*OR* = 1.345, 95%*CI*: 1.046–1.730), having daily outdoor activity of no less than 2 h (*OR* = 1.348, 95%*CI*: 1.063–1.711), holding the view that sugar-sweetened beverages pose no health risks (*OR* = 1.916, 95%*CI*: 1.561–2.352), and having caregivers who regularly consumed fruit and vegetable juices and related beverages (*OR* = 1.716, 95%*CI*: 1.404–2.097) were all correlated with elevated odds of excessive sugar intake (all *p* < 0.05). Gender, underweight, obesity, and caregiver's education showed no independent associations (all *p* > 0.05).

**Table 5:** Multivariate binary logistic regression for excessive daily free sugar intake (≥25 g) among school-aged children in Beijing, China.

Independent Variable	*B*	*S.E*	Wald χ2	*OR*(95%*CI*)	*P*-value
Residence areas (ref: Central urban)					
Outer urban	0.381	0.127	8.935	1.464 (1.140–1.879)	0.003
sSuburban	0.367	0.127	8.373	1.443 (1.126–1.849)	0.004
Gender (ref: Girls)					
Boys	0.174	0.103	2.860	1.190 (0.793–1.457)	0.091
Grade (ref: Primary school)					
Middle school	0.661	0.133	24.820	1.936 (1.493–2.511)	< 0.001
High school	0.353	0.139	6.478	1.423 (1.085–1.868)	0.011
Weight type status (ref: Normal)					
Underweight	0.135	0.214	0.399	1.145 (0.752–1.743)	0.528
Overweight	0.297	0.128	5.348	1.345 (1.046–1.730)	0.021
Obese	0.131	0.130	1.015	1.140 (0.884–1.471)	0.314
Daily outdoor activity time (ref:<2 h/day)					
≥2 h/day	0.299	0.122	6.053	1.348 (1.063–1.711)	0.014
Perception of sugar sweetened beverages harm to health (ref: Harmful)					
Harmless	0.650	0.105	38.629	1.916 (1.561–2.352)	< 0.001
Caregiver's education (ref: High school or below)					
College or above	0.001	0.105	0.000	1.001 (0.815–1.230)	0.989
Caregivers' consumption of fruit and vegetable juices and related beverages (ref: Non-consumption)					
Consumption	0.540	0.102	27.874	1.716 (1.404–2.097)	< 0.001

### 3.5 Consumption characteristics of fruit and vegetable juices and related beverages in consumers

#### 3.5.1 Daily intake of fruit and vegetable juices and related beverages and corresponding free sugar intake in consumers

Table 6 presents the daily intake of fruit and vegetable juices and related beverages and corresponding free sugar intake among consuming children in Beijing, China. Overall, the median daily intake of juice beverages and related beverages was 107.14 g (57.14, 214.29 g), with a median free sugar intake of 11.36 g (6.06, 22.71 g). Significant differences in both intake measures were observed across residence area, gender, grade, ethnicity, household registration type, myopia status, perceived harm of sugar-sweetened beverages, food label checking behavior, daily outdoor activity duration, caregiver's education level, and caregiver's fruit and vegetable juices and related beverages consumption (all *p* < 0.05). In general, higher intakes were found in children living in outer urban and suburban areas, boys, middle and high school students, non-Han children, those with agricultural household registration, children with myopia, those who perceived sugar-sweetened beverages as harmless, those who never checked food labels, those with ≥2 h of daily outdoor activity, and those whose caregivers had lower educational levels or consumed juice beverages. No significant differences were detected in fruit and vegetable juices and related beverages and corresponding free sugar intake by weight status, dental caries, attitudes toward juice replacing whole fruits and vegetables, number of children in the family, or caregivers' perceptions of juice substitution (all *p* > 0.05).

**Table 6:** Daily intake of fruit and vegetable juices and related beverages and corresponding free sugar intake among consumers in Beijing, China.

Characteristics	Daily intake of fruit and vegetable juices and related beverages *M*(*P*_25_, *P*_75_)	Daily free sugar intake *M*(*P*_25_, *P*_75_)	χ^2^	*P*-value
Total	107.14 (57.14, 214.29)	11.36 (6.06, 22.71)		
Residence area			26.113	< 0.001
Central urban	92.86 (42.86, 185.71)	9.84 (4.54, 19.69)		
Outer urban	121.43 (57.14, 242.86)	12.87 (6.06, 25.74)		
Suburban	114.29 (71.43, 228.57)	12.11 (7.57, 24.23)		
Gender			−3.280	0.001
Boys	114.29 (64.29, 242.86)	12.11 (6.81, 25.74)		
Girls	107.14 (47.14, 207.14)	11.36 (5.00, 21.96)		
Grade			92.696	< 0.001
Primary school	71.43 (35.71, 190.71)	7.57 (3.79, 20.22)		
Middle school	128.57 (71.43, 250.00)	13.63 (7.57, 26.50)		
High school	121.43 (71.43, 219.64)	12.87 (7.57, 23.28)		
Ethnicity			−2.598	0.009
Han	107.14 (57.14, 214.29)	11.36 (6.06, 22.71)		
Others	142.86 (71.43, 228.57)	15.14 (7.57, 24.23)		
Household registration type			−2.293	0.022
Agricultural	128.57 (71.43, 242.86)	13.63 (7.57, 25.74)		
Urban	107.14 (57.14, 214.29)	11.36 (6.06, 22.71)		
Weight type status			5.248	0.155
Normal	107.14 (57.14, 210.36)	11.36 (6.06, 22.30)		
Underweight	89.29 (50.00, 205.36)	9.46 (5.30, 21.77)		
Overweight	119.29 (64.29, 250.00)	12.64 (6.81, 26.50)		
Obese	114.29 (51.07, 228.57)	12.11 (5.41, 24.23)		
Dental caries			−1.533	0.125
No	112.14 (57.14, 214.86)	11.89 (6.06, 22.71)		
Yes	107.14 (50.00, 207.14)	11.36 (5.30, 21.96)		
Myopia			−2.707	0.007
No	100.00 (42.86, 207.14)	10.60 (4.54, 21.96)		
Yes	114.29 (57.14, 214.29)	12.11 (6.06, 22.71)		
Perception of sugar sweetened beverages harm to health			−7.759	< 0.001
Harmless	142.86 (71.43, 285.71)	15.14 (7.57, 30.29)		
Harmful	100.00 (42.86, 202.86)	10.60 (4.54, 21.50)		
Perception of fruit and vegetable juices and related beverages replacing whole fruits and vegetables			−0.293	0.770
Replaceable	107.14 (42.86, 232.14)	11.36 (4.54, 24.61)		
Non-replaceable	107.14 (57.14, 214.29)	11.36 (6.06, 22.71)		
Checking food labels before purchase			−1.979	0.048
Never check	114.29 (57.14, 225.71)	12.11 (6.06, 23.93)		
Check	107.14 (50.00, 214.29)	11.36 (5.30, 22.71)		
Daily outdoor activity time			−2.831	0.005
< 2 h/day	107.14 (57.14, 214.29)	11.36 (6.06, 22.71)		
≥2 h/day	128.57 (64.29, 257.14)	13.63 (6.81, 27.26)		
Number of children in family			−0.611	0.541
≥2	107.14 (57.14, 214.29)	11.36 (6.06, 22.71)		
1	114.29 (57.14, 214.29)	12.11 (6.06, 22.71)		
Caregiver's education			−2.899	0.004
High school or below	121.43 (64.29, 221.43)	12.87 (6.81, 23.47)		
College or above	107.14 (50.00, 214.29)	11.36 (5.30, 22.71)		
Caregivers' perception of fruit and vegetable juices and related beverages replacing whole fruits and vegetables			−1.615	0.106
Replaceable	142.86 (71.43, 250.00)	15.14 (7.57, 26.50)		
Non-replaceable	107.14 (57.14, 214.29)	11.36 (6.06, 22.71)		
Caregivers' consumption of fruit and vegetable juices and related beverages			−2.788	0.005
Non-consumption	107.14 (47.86, 207.14)	11.36 (5.07, 21.96)		
Consumption	114.29 (57.14, 228.57)	12.11 (6.06, 24.22)		

#### 3.5.2 Univariate analysis of excessive daily free sugar intake from fruit and vegetable juices and related beverages in consumers

Table 7 shows the prevalence of excessive daily free sugar intake (≥25 g) from fruit and vegetable juices and related beverages among consuming school-age children in Beijing. Overall, 518 consumers (22.5%) had free sugar intake exceeding 25 g per day. Significant differences in the prevalence of excessive intake were observed by residence area, gender, grade, weight status, perceived harm of sugar-sweetened beverages, daily outdoor activity time, and caregiver's juice consumption (all *p* < 0.05). Higher proportions of excessive intake were found in outer urban and suburban areas, among boys, middle and high school students, overweight children, those who perceived sugar-sweetened beverages as harmless, those with ≥2 h daily outdoor activity, and those whose caregivers consumed juice beverages. In contrast, ethnicity, household registration type, dental caries, myopia, beliefs about juice replacing whole fruits and vegetables, food label checking, number of children in the household, caregiver's education, and caregivers' substitution beliefs were not significantly associated with excessive free sugar intake (all *p* > 0.05).

**Table 7:** The prevalence of excessive daily free sugar intake (≥25 g) from fruit and vegetable juices and related beverages among consumers in Beijing, China.

Characteristics	< 25 g/day, *n*(%)	≥25 g/day, *n*(%)	χ^2^	*P*-value
Total	1,788 (77.5)	518 (22.5)		
Residence area			14.316	0.001
Central urban	631 (82.1)	138 (17.9)		
Outer urban	549 (74.3)	190 (25.7)		
Suburban	608 (76.2)	190 (23.8)		
Gender			10.943	0.001
Boys	850 (74.6)	289 (25.4)		
Girls	938 (80.4)	229 (19.6)		
Grade			25.212	< 0.001
Primary school	531 (83.8)	103 (16.2)		
Middle school	629 (72.8)	235 (27.2)		
High school	628 (77.7)	180 (22.3)		
Ethnicity			0.005	0.944
Han	1,631 (77.6)	472 (22.4)		
Others	157 (77.3)	46 (22.7)		
Household registration type			2.909	0.088
Agricultural	187 (73.3)	68 (26.7)		
Urban	1,601 (78.1)	450 (21.9)		
Weight type status			9.499	0.023
Normal	1,009 (79.6)	259 (20.4)		
Underweight	118 (79.7)	30 (20.3)		
Overweight	313 (73.1)	115 (26.9)		
Obese	348 (75.3)	114 (24.7)		
Dental caries			2.377	0.123
No	1,192 (76.6)	364 (23.4)		
Yes	596 (79.5)	154 (20.5)		
Myopia			0.759	0.384
No	543 (78.7)	147 (21.3)		
Yes	1,245 (77.0)	371 (23.0)		
Perception of sugar sweetened beverages harm to health			51.723	< 0.001
Harmless	425 (67.4)	206 (32.6)		
Harmful	1,363 (81.4)	312 (18.6)		
Perception of fruit and vegetable juices and related beverages replacing whole fruits and vegetables			0.789	0.374
Replaceable	173 (75.2)	57 (24.8)		
Non-replaceable	1,615 (77.8)	461 (22.2)		
Checking food labels before purchase			1.713	0.191
Never check	709 (76.2)	222 (23.8)		
Check	1,079 (78.5)	296 (21.5)		
Daily outdoor activity time			9.549	0.002
< 2 h/day	1,487 (78.8)	400 (21.2)		
≥2 h/day	301 (71.8)	118 (28.2)		
Number of children in family			1.261	0.262
≥2	700 (76.3)	217 (23.7)		
1	1,088 (78.3)	301 (21.7)		
Caregiver's education			1.885	0.170
High school or below	679 (76.0)	214 (24.0)		
College or above	1,109 (78.5)	304 (21.5)		
Caregivers' perception of fruit and vegetable juices and related beverages replacing whole fruits and vegetables			0.228	0.633
Replaceable	45 (75.0)	15 (25.0)		
Non-replaceable	1,743 (77.6)	503 (22.4)		
Caregivers' consumption of fruit and vegetable juices and related beverages			4.723	0.030
Non-consumption	691 (80.0)	173 (20.0)		
Consumption	1,097 (76.1)	345 (23.9)		

#### 3.5.3 Multivariate binary logistic regression for excessive daily free sugar intake (≥25 g) in consumers

Table 8 presents the results of multivariate binary logistic regression analysis examining factors associated with excessive daily sugar intake (≥25 g) from fruit and vegetable juices and related beverages among consuming school-aged children. Further analyses identified the following correlates for excessive sugar intake in this subgroup: residence in outer urban areas (*OR* = 1.467, 95%*CI*: 1.138–1.892) or suburban areas (*OR* = 1.426, 95%*CI*: 1.108–1.837), male gender (*OR* = 1.289, 95%*CI*: 1.046–1.589), middle school attendance (*OR* = 1.643, 95%*CI*: 1.254–2.152), overweight status (*OR* = 1.341, 95%*CI*: 1.031–1.743), daily outdoor activity of no less than 2 h (*OR* = 1.297, 95%*CI*: 1.011–1.664), the perception of sugar sweetened beverages to health as harmless (*OR* = 1.900, 95%*CI*: 1.532–2.358), and caregivers' consumption of fruit and vegetable juices and related beverages (*OR* = 1.261, 95%*CI*: 1.021–1.556) (all *p* < 0.05). High school, underweight, and obesity showed no independent associations (all *p* > 0.05).

**Table 8:** Multivariate binary logistic regression for excessive daily free sugar intake (≥25 g) in consumers.

Independent Variable	*B*	*S.E*	Wald χ2	*OR*(95%*CI*)	*P*-value
Residence areas (ref: Central urban)					
Outer urban	0.383	0.130	8.761	1.467 (1.138–1.892)	0.003
Suburban	0.355	0.129	7.588	1.426 (1.108–1.837)	0.006
Gender (ref: Girls)					
Boys	0.254	0.107	5.655	1.289 (1.046–1.589)	0.017
Grade (ref: Primary school)					
Middle school	0.496	0.138	12.991	1.643 (1.254–2.152)	< 0.001
High school	0.243	0.142	2.931	1.276 (0.965–1.685)	0.087
Weight type status (ref: Normal)					
Underweight	0.063	0.223	0.081	1.065 (0.689–1.648)	0.776
Overweight	0.293	0.134	4.783	1.341 (1.031–1.743)	0.029
Obese	0.158	0.135	1.365	1.171 (0.899–1.526)	0.243
Daily outdoor activity time (ref:<2 h/day)					
≥2 h/day	0.260	0.127	4.195	1.297 (1.011–1.664)	0.041
Perception of sugar sweetened beverages harm to health (ref: Harmful)					
Harmless	0.642	0.110	34.016	1.900 (1.532–2.358)	< 0.001
Caregivers' consumption of fruit and vegetable juices and related beverages (ref: Non-consumption)					
Consumption	0.232	0.107	4.652	1.261 (1.021–1.556)	0.031

## 4 Discussion

In this cross-sectional study of 3,370 school-aged children in Beijing, the consumption rate of fruit and vegetable juices and related beverages reached 68.4%. The median daily intake stood at 57.14 mL, with a median free and added sugar contribution of 6.06 g. A total of 15.4% of children had a daily sugar intake above the 25 g threshold. Intake of free and added sugars from these beverages was markedly lower than figures from a study in Changsha, which recorded a daily free sugar intake of 53.1 g among local adolescents (23). The consumption rate for fruit and vegetable beverages was 58.9%, evidently higher than the 43.4% for fruit and vegetable juices. Both figures were also considerably higher than data from Beijing school-aged children in 2022, namely 24.6% for fruit and vegetable beverages and 32.3% for fruit and vegetable juices (15). In addition, fruit and vegetable beverages saw a larger rise in consumption rate compared with fruit and vegetable juices. These observations collectively suggest that school-aged children exhibit a strong preference for both fruit and vegetable juices and fruit and vegetable beverages, with a particularly pronounced inclination toward the latter. In our study, median free/added sugars intake reached 107.14 g, with 22.5% showing excessive sugar intake among consumers. These findings highlight that fruit and vegetable juices and related beverages serve as a major source of free sugars in children's daily diets. Of the 198,285 study participants who all completed at least one online diet questionnaire, 32.8% drank sugar-sweetened beverages, 20.6% drank artificially sweetened beverages and 52.2% drank fruit and vegetable juices (24). Although the findings of the present study were considerably lower than those reported in European countries, the United Kingdom, Switzerland, the United States and other countries, they confirm that school-aged children in China, like those in other regions and countries, widely consume fruit-based beverages, which contribute substantially to their daily sugar intake (24–27). The World Health Organization recommends reducing the intake of free sugars to less than 5% (< 25 g)of total daily energy intake ideally, thus including sugars naturally present in fruit and juices in the category of sugars whose consumption should be reduced (9).

Multivariate regression analysis demonstrated that school-aged children living in outer urban and suburban areas tended to have higher odds of excessive free sugar intake from fruit and vegetable juices and related beverages, compared with children residing in central urban areas. Boys were also more likely to exceed the sugar intake threshold than girls. Similarly, students from middle and high schools presented higher odds than primary school students, and children spending no less than 2 h on outdoor activities each day had elevated odds relative to those with shorter outdoor activity time. These observations have noted that children in outer urban and suburban areas, boys and older students generally engage in more physical activity and sweat more, which may increase their demand for beverages (28–30). This group also has easier access to fruit and vegetable juices and related products, especially fruit and vegetable beverages (31, 32). Additionally, older children have greater autonomy over food selection, which may be linked to higher intake of such beverages (33, 34). As this was a cross-sectional study, causal relationships cannot be established based on the observed associations, particularly the link between overweight status and juice intake. This study also found overweight children were most likely to have excessive sugar exposure from these drinks. This finding correlates with existing evidence reporting an association between high sugar intake and elevated body weight as well as overweight or obesity among children (35–37). Meanwhile, children who regarded sugar-sweetened beverages as harmless had higher odds of excessive sugar intake than those who acknowledged potential health hazards. Adequate nutrition literacy helps people recognize health risks and adopt healthier dietary habits, which is consistent with prior findings (38, 39). The above associations may be partly explained by insufficient knowledge about the health risks of sugary drinks among school-aged children (38, 39). A large number of children mistakenly regard fruit and vegetable juices and related beverages as healthy options. They fail to recognize that fruit and vegetable beverages belong to high added-sugar drinks, and overlook the high content of free sugars in pure fruit and vegetable juices (40, 41). Collectively, these results suggest a pressing need to carry out targeted interventions for school-aged children. Relevant initiatives should focus on disseminating knowledge about the adverse effects of excessive free sugar intake, improving skills to read nutrition labels, and helping children distinguish pure juices from juice beverages. It is essential to raise awareness that fruit and vegetable juices cannot replace whole fruits, vegetables or plain water, and that frequent or overconsumption of these drinks may compromise physical health.

In addition, children whose caregivers consumed fruit and vegetable juices and related beverages were more likely to have excessive sugar intake. This result supports the well-documented family behavioral modeling effect, which indicates that caregivers' dietary habits are closely linked to children's beverage preferences and intake patterns. It further highlights the value of family centered interventions to reduce children's excessive sugar consumption (42, 43). Many children and their caregivers lack adequate awareness of the high free sugar content in fruit and vegetable juices, and struggle to differentiate pure juices from juice beverages loaded with added sugars. Influenced by product packaging and marketing featuring fruits and vegetables, both children and caregivers often misjudge these products as healthy choices, and hold the misconception that such drinks can substitute for whole produce or plain water.

Therefore, targeted interventions for caregivers are also urgently required, which can be implemented from three key aspects (9, 11, 14). First, interventions should help caregivers correctly interpret food labels and make rational choices for beverages, with a focus on distinguishing pure fruit and vegetable juices from juice beverages. Pure juices contain considerable free sugars but retain some nutrients from raw fruits and vegetables. By contrast, juice beverages are typical sugar-sweetened products with abundant added sugars, artificial sweeteners and other additives, and their consumption should be discouraged. Second, caregivers need to understand the potential health concerns associated with high free and added sugar levels in these drinks. Third, it should be emphasized that fruit and vegetable juices cannot take the place of whole fruits, vegetables or plain water in children's daily diets. Although pure juices can serve as a temporary alternative when fresh produce is unavailable, their high sugar content makes them unsuitable for regular or heavy consumption. Frequent juice drinking may also reduce chewing frequency, which may be associated with suboptimal development of oral muscles and related physical functions in children.

This study provides updated population-based evidence on the consumption of fruit and vegetable juices and related beverages and associated factors among Chinese school-aged children. However, several limitations should be noted. First, the study population was restricted to students in Grade 3 of primary school, Grade 7 of junior high school, and Grade 11 of senior high school in Beijing, China. Future studies should expand the sampling scope to cover all grades of school-aged children to comprehensively reflect regional consumption patterns and assess health risks related to excessive free sugar intake from such beverages. Second, this study adopted a cross-sectional design, which precludes causal inference. Furthermore, beverage consumption was based on self-reported data, which may involve potential reporting bias. This reporting bias mainly manifests in two aspects. On the one hand, children and their caregivers tend to underreport frequent or excessive intake of sugary beverages out of awareness of relevant health guidance, leading to an underestimation of actual consumption volume and sugar exposure level. On the other hand, respondents may also mistakenly recall occasional intake as regular consumption, which could moderately overestimate the overall consumption rate. The impact may skew the distribution of core consumption indicators. Future studies may combine self-reported questionnaires with objective data and behavioral records from multiple sources to mutually correct biases. Data can be collected on different days and in varied settings such as weekdays vs. weekends, as well as school and home environments. Meanwhile, consumption transaction records can be incorporated to reduce the overall impact of recall bias.

## 5 Conclusion

In conclusion, fruit and vegetable juices and related beverages are highly prevalent among school-aged children in Beijing, more than 15% of children exceeded the recommended free sugar intake from these beverages, and the proportion reached over 20% among regular consumers, which may contribute substantially to excessive free sugar intake. Residential area, school stage, weight status, outdoor activity time, perception of sugar-sweetened beverages, and caregiver consumption were associated factors. These findings highlight the urgent need for targeted interventions, including nutrition education for children and caregivers, promoting the consumption of whole fruit and vegetables instead of juices, improving product labeling, and reducing sugar content in commercially available beverages. Such strategies are critical to reduce excessive free sugar intake from fruit and vegetable juices and related beverages and protect the healthy growth of school-aged children.

## Data Availability

The original contributions presented in the study are included in the article/supplementary material, further inquiries can be directed to the corresponding authors.
